# The Application of Hollow Carbon Nanofibers Prepared by Electrospinning to Carbon Dioxide Capture

**DOI:** 10.3390/polym13193275

**Published:** 2021-09-25

**Authors:** Yu-Chun Chiang, Wei-Ting Chin, Chih-Cheng Huang

**Affiliations:** 1 Department of Mechanical Engineering, Yuan Ze University, Taoyuan 320, Taiwan; s1060806@mail.yzu.edu.tw (W.-T.C.); s1060807@mail.yzu.edu.tw (C.-C.H.); 2 Fuel Cell Center, Yuan Ze University, Taoyuan 320, Taiwan

**Keywords:** electrospinning, hollow carbon nanofibers, carbon dioxide, adsorption

## Abstract

Coaxial electrospinning has been considered a straightforward and convenient method for producing hollow nanofibers. Therefore, the objective of this study was to develop hollow activated carbon nanofibers (HACNFs) for CO_2_ capture in order to reduce emissions of CO_2_ to the atmosphere and mitigate global warming. Results showed that the sacrificing core could be decomposed at carbonization temperatures above 900 °C, allowing the formation of hollow nanofibers. The average outer diameters of HACNFs ranged from 550 to 750 nm, with a shell thickness of 75 nm. During the carbonization stage, the denitrogenation reactions were significant, while in the CO_2_ activation process, the release of carbon oxides became prominent. Therefore, the CO_2_ activation could increase the percentages of N=C and quaternary N groups. The major nitrogen functionalities on most samples were O=C–NH and quaternary N. However, =C and quaternary N groups were found to be crucial in determining the CO_2_ adsorption performance. CO_2_ adsorption on HACNFs occurred due to physical adsorption and was an exothermic reaction. The optimal CO_2_ adsorption performance was observed for HACNFs carbonized at 900 °C, where 3.03 mmol/g (1 atm) and 0.99 mmol/g (0.15 atm) were measured at 25 °C. The degradation of CO_2_ uptakes after 10 adsorption−desorption cyclic runs could be maintained within 8.9%.

## 1. Introduction

Anthropogenic carbon dioxide (CO_2_) emissions to the atmosphere have resulted in several global environmental impacts, such as global climate change and global warming. Many countries have announced pledges to achieve net-zero emissions over the coming decades to bring global energy-related CO_2_ emissions to net zero by 2050. Currently, there is high interest in studies of CO_2_ capture by adsorption on porous carbonaceous or non-carbonaceous materials [1,2,3]. However, the development of commercially available porous adsorbents for CO_2_ capture is still in progress. The volume of pores less than 0.7 nm in size and the presence of surface nitrogen groups have been regarded as the most important factors determining good CO_2_ adsorbents [4,5,6,7,8]. Several porous materials have been intensively investigated as potential candidates for CO_2_ capture, such as activated carbons, activated carbon fibers, carbon nanofibers, zeolites, metal organic frameworks, zeolitic imidazolate frameworks, silica, and carbon nanotubes. Among these porous materials, carbon fibers have attracted much attention due to their unique properties, particularly when they are reduced to the nanoscale [9,10,11]. Several process techniques have been applied to synthesize polymer nanofibers, such as drawing [12,13], template synthesis [14,15], phase separation [16], self-assembly [17,18], and electrospinning [19,20]. Compared with other methods, electrospinning is acknowledged as a highly versatile, relatively simple, inexpensive, and efficient method for processing polymers into continuous fibers with diameters ranging from nanometers to submicrons [9,21] or mats structures [22,23].

A basic electrospinning setup comprises a high-voltage power supply, a syringe container, a needle nozzle, and a counter electrode collector [24]. Polymer solutions, especially thermoplastic polymers, are often used as spinning materials. Electrospinning processes involve a complex interplay of surfaces, shapes, rheology, and electrical charge [21,25,26,27]. The diameter of electrospun nanofibers is highly influenced by the concentration of the polymer, the solution viscosity, and the molecular conformation [26,28]. The electrospinning of polymer solutions only forms polymer droplets in the dilute concentration range because of an insufficient chain overlap. As the concentration is increased, beaded fibers and further uniform fibers can be observed sequentially [29]. Above a minimum concentration, a fibrous structure is stabilized, while the fiber diameter increases with the molecular weight and concentration [28]. For the polymers with higher molecular weights, uniform fibers can be formed at a lower polymer concentration compared with those with lower molecular weights [30]. Therefore, the stabilization of fibrous structures is subject to intermolecular entanglements [31].

When a spinneret (fiber generator) containing two or more channels is employed for feeding different polymer solutions, several types of nanofibers with a special cross-sectional configuration, such as core/shell [32], hollow [27], side by side [33], or crimped structure [33], can be produced, depending on the spinneret structure. In addition, blending a sacrificial polymer into another polymer solution can lead to the formation of a porous structure [34]. Coaxial electrospinning is a widely used method for obtaining hollow nanofibers, in which the single spinneret is replaced by a coaxial spinneret. Using this configuration, co-electrospinning immiscible and miscible pairs of polymer solutions forms nanofibers with core/shell structures [35]. Subsequently, the core material can be removed by thermal decomposition [35,36] or extraction with mineral oil or a solvent [37]. It has been suggested that the core-polymer capillary must protrude ~1 mm below the end of the shell capillary to produce a good Taylor cone at the end of the nozzle [35,38]. Good morphologies of polyacrylonitrile (PAN)/poly (methyl methacrylate) (PMMA) nanofibers were obtained from coaxial nozzle electrospinning at 15 kV. If the applied electrical voltage was greater than 20 kV, the extraction rate of the polymer solution was too fast to evaporate the solvent. Therefore, wet nanofibers were molten together as a bundle of fibers. For an applied voltage of 10 kV, the injection was retarded for a period until the electrostatic force was strong enough to inject the solution jet, and some of the nanofibers were molten together to form a bundle morphology [38].

Due to their high specific surface areas, high surface area to volume ratios, easy functionalization, superior mechanical properties, outstanding flexibility, controllability in fiber diameter, surface morphology, fibrous structure [24], etc., hollow carbon nanofibers have significant potential for application in domains as diverse as drug delivery [39] and as a scaffold for tissue engineering [40], in the capacitive deionization process for water treatment [41], and in energy [42,43,44,45,46].

Electrospinning techniques have become a straightforward and convenient method for producing continuous and porous carbon nanofibers [20]. However, they have very few applications in gas adsorption. Therefore, the objective of this study was to prepare hollow activated carbon nanofibers (HACNFs) in which polyacrylonitrile (PAN) was used as the shell polymer and poly (methyl methacrylate) (PMMA) was selected as the sacrificing core. Thermal treatments have been widely adopted for improving the properties of carbon nanofibers [47]. By means of coaxial electrospinning followed by stabilization, carbonization, and activation, HACNFs were formed. Several properties of the HACNFs were characterized, and the CO_2_ adsorption on the HACNFs was discussed.

## 2. Materials and Methods

### 2.1. Fabrication of Hollow Electrospun Nanofibers

In this study, hollow carbon nanofibers were synthesized using a coaxial electrospinning process in which polyacrylonitrile (PAN, Mw = 150 kDa, Sigma-Aldrich, Burlington, MA, USA) was selected as the shell polymer and poly (methyl methacrylate) (PMMA, Mw = 120 kDa, Sigma-Aldrich) was chosen as the sacrificing core. Firstly, PAN and PMMA were dissolved in N, N-dimethylacetamide (DMAc, Sigma-Aldrich, Burlington, MA, USA) by magnetic stirring at 60 °C for 24 h to form homogeneous polymer solutions in concentrations of 10 wt.% and 30 wt.%, respectively. The core–shell fibers were fabricated using a coaxial spinneret system in an electrospinning unit (FES-COE, Falco Tech Enterprise Co., Ltd., New Taipei City, Taiwan). The outer needle (18 gauge) had an inner diameter of 0.96 mm and the inner needle (21 gauge) had inner and outer diameters of 0.52 and 0.82 mm, respectively. The electrospinning was conducted under a work voltage of 15 kV onto a metal drum collector (∅ 10 cm) covered with aluminum foil and rotated at 300 rpm, and a work distance (a tip to collector distance) of 15 cm was used. The feed rates of the shell and core solutions were 1.0 and 0.5 mL/h [36], respectively, and the process was propelled by two syringe pumps (NE-1000, New Era Pump Systems, Inc., Farmingdale, NY, USA).

The collected core–shell nanofibers were stabilized in a furnace with an air atmosphere from room temperature to 280 °C with a heating rate of 1 °C/min; then, the temperature was held at 280 °C for 12 h to finish the cyclization and dehydrogenation reactions and convert PAN from a thermoplastic to non-plastic compound [48,49]. This is a significant step in fabricating ACNFs due to the formation of a conjugated ladder structure [50]. The stabilized nanofibers were cooled down to room temperature and then carbonized at 900, 1000, or 1100 °C with a heating rate of 5 °C/min and maintained for 1 h under flowing nitrogen with a flow of 100 sccm in a tubular furnace [43]. After carbonization, the activation of the samples was accomplished by raising the temperature to 850 °C at a rate of 10 °C/min in a nitrogen atmosphere. The activation agent was a pure CO_2_ gas, which was switched in when a temperature of 850 °C was reached in a tubular furnace and maintained for 1 h with a flow rate of 100 sccm. The samples carbonized at 900, 1000, or 1100 °C were labeled as C900, C1000, or C1100, respectively. After the carbonized samples were further activated, they were denoted as A900–850, A1000−850, or A1100−850.

### 2.2. Characterizations

Field emission scanning electron microscopy (FESEM) was utilized to observe the morphology of the samples using a scanning electron microscope (S−4800, Hitachi, Krefeld, Germany). The elemental compositions in the samples were analyzed using elemental analysis (EA), where the weight percent (wt.%) of the C, H, and N elements was measured by an elemental analyzer (Elementar vario EL cube, Langenselbold, Germany). Fourier transform infrared (FTIR) spectroscopy is a technique used to obtain an infrared spectrum of absorption or emission of a solid. However, FTIR could not give much information about chemical structures for carbonaceous materials [51] due to the strong absorption of infrared light by the carbon. Therefore, FTIR with attenuated total reflectance (ATR) mode was used to obtain the FTIR spectra of the samples. The FTIR spectra were acquired using a FTIR spectrometer (Perkin Elmer, Spectrum 100, Waltham, MA, USA) in a range of wavenumbers from 4000 to 650 cm^−1^ at a resolution of 4 cm^−1^. The number and type of functional groups present on the surface of the samples were analyzed using X-ray photoelectron spectroscopy (XPS), in which information on the elements within a few nanometers of the sample surface could be obtained. The XPS spectra were collected using a spectrophotometer (PHI 5000 VersaProbe II, ULVAC-PHI, Kanagawa, Japan), in which a scanning X-ray monochromator (Al Anode, hν = 1401 eV) was used. For calibration purposes, the C 1 s electron binding energy (BE) that corresponds to graphitic carbon was set at 285 eV. A nonlinear least squares curve-fitting program (XPSPEAK software, version 4.1, The Chinese University of Hong Kong, Hong Kong, China) was employed in the deconvolution of the XPS spectra. N_2_ adsorption–desorption isotherms measured at –196 °C using an ASAP 2020 accelerated surface area and porosimetry system (Micromeritics, Norcross, GA, USA) were investigated in order to find the surface structures of the samples. All samples were degassed at 350 °C for 24 h to eliminate the interference of the moisture and trace gases prior to the adsorption measurements. The specific surface areas (SSAs) of the samples were determined at relative pressures from 0.05 to 0.3 using the Brunauer–Emmett–Teller (BET) method. The micropore (<2.0 nm) surface area (S_mi_) was derived using the t-plot method. The single point total pore volume (V_t_) was obtained at a relative pressure of approximately 0.99. The mesopore volume (V_me_), micropore volume (V_mi_), and ultramicropore (<0.7 nm) volume (V_<0.7 nm_) were obtained by adopting a non-local density functional theory (NLDFT) model, heterogeneous surface−2 dimensional−NLDFT (HS−2D−NLDFT), where V_<0.7 nm_ is part of V_mi_. The median pore width (d_HK_) was obtained using the Horváth–Kawazoe (HK) method. The pore size distribution curves were also inferred from the HS−2D−NLDFT model. 

### 2.3. CO_2_ Adsorption Experiments

The Micromeritics ASAP 2020 system was also used to measure the CO_2_ adsorption isotherms on the activated samples at 25, 40, and 55 °C under a CO_2_ pressure of less than 123 kPa. The samples (approximately 0.05 g) were outgassed at 350 °C for 24 h for the removal of adsorbed contaminants prior to the measurement. The equilibration interval for each pressure point was set as 45 s. A chiller dewar (Micromeritics) was utilized to replace the traditional dewar, which could be connected with a circulating water bath thermostat for the temperature control during the CO_2_ adsorption process.

The Freundlich equation [52], one of the most commonly used equations for adsorption isotherms, was adopted for the model fitting of the adsorption experimental data. The Freundlich adsorption isotherm is an empirical equation that assumes heterogeneous adsorption due to the diversity of adsorption sites, as shown in Equation (1):

(1) $$q_{e}=K_{F}P^{1/n},$$

where $q_{e}$ (mmol/g) is the equilibrium adsorption capacity, $K_{F}$ ((mmol/g) (1/kPa)^1/n^) is the Freundlich adsorption coefficient, *P* (kPa) is the gas pressure, and *n* is a constant indicating the isotherm curvature. The parameter *n* is usually greater than unity, and the larger the value of *n* is the more favorable the adsorption is. In general, the values of parameters *n* and $K_{F}$ both decrease with increasing temperature.

**Scheme 1:**
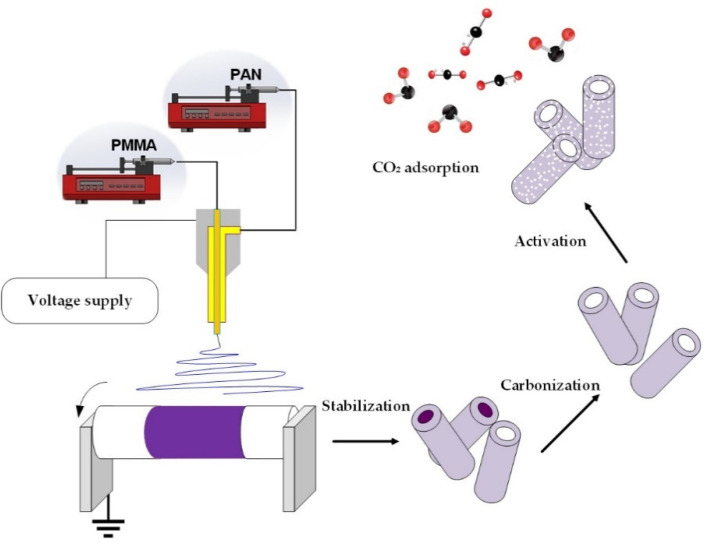
Schematic illustration of this study, including electrospinning, stabilization, carbonization, activation, and CO_2_ adsorption.

The isosteric heat of adsorption ($Q_{st}$) measures the change in enthalpy when adsorbate molecules are adsorbed from the bulk gas phase to the adsorbed phase [52]. It represents the interactions between the adsorbate molecules and the adsorbent lattice atoms and provides a measure of energetic heterogeneity for the gas–solid interfaces [53]. The Clausius–Clapeyron equation, as shown in Equation (2), was used to calculate the values of $Q_{st}$:

(2) $$-\frac{Q_{st}}{R}=\left(\frac{d\ln P}{d\;\frac{1}{T}}\right),$$

where $Q_{st}$ (kJ/mol) is the isosteric heat of adsorption, *R* (=8.314 J/mol/K) is the gas constant, *P* (kPa) is the CO_2_ pressure, and *T* (K) is the adsorption temperature. A schematic illustration of this study is shown in Scheme 1, which includes electrospinning, stabilization, carbonization, activation, and CO_2_ adsorption.

## 3. Results and Discussion

### 3.1. Field Emission Scanning Electron Microscopy (FESEM) Images

**Figure 1:**
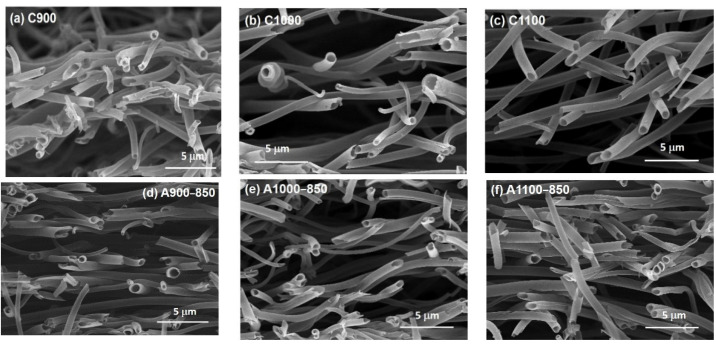
Field emission scanning electron microscopy (FESEM) images of the samples: (**a**) C900; (**b**) C1000; (**c**) C1100; (**d**) A900−850; (**e**) A1000−850; and (**f**) A1100−850.

Figure 1a–c show the FESEM micrographs of the cross-sectional areas for all the carbonized samples. These images clearly evidence the formation of hollow tubular structures. The shell material (PAN) was converted into a turbostratic carbon structure by thermal treatment at temperatures of 900 °C or above, whereas the sacrificial core component (PMMA) was decomposed. After activation, the samples could hold the hollow structures (Figure 1d–f). If a sacrificial core is decomposed at an early stage of the thermal treatment, the shell polymer of HACNFs will be subjected to severe shrinkage without any support; thus, the shapes of HACNFs may be distorted [54]. Although the thermal decomposition temperature of PMMA was below 300 °C [55], the HACNFs prepared in this study did not appear to be out of shape, as seen from Figure 1. It was expected that the use of a slow heating rate (1 °C/min) during the stabilization step could prevent the sacrificial core from evaporating too fast and thus preserve the shape of the shell component. Due to its immiscibility with PAN, PMMA was thought to be a suitable material for use as the sacrificial core for fabricating HACNFs. All nanofibers were randomly aligned along the winding direction of the drum collector. The outer and interior surfaces of hollow nanofibers were relatively smooth. The outer diameters of C900, C1000, and C1100 were approximately 725, 758, and 842 nm, respectively, with a shell thickness of 100 nm. For the activated samples, the outer diameters of A900−850, A1000−850, and A1100−850 were approximately 550, 605, and 750 nm, respectively, with a shell thickness of about 75 nm. 

### 3.2. Elemental Analysis (EA)

The elemental compositions of C, H, and N atoms in the bulk phase of the samples are shown in Table 1. The most abundant nitrogen content was observed in C900 (8.0 wt.%), in which the nitrogen was derived from the C≡N bonding in a PAN precursor. As the carbonization temperature increased, the nitrogen content in the carbonized samples reduced rapidly. This implied that the carbonization process involved successive denitrogenation reactions, eventually resulting in partially graphitic structures. The activated samples had a similar behavior—i.e., the nitrogen content decreased with an increase in the carbonization temperature. Compared with the pair samples—i.e., the samples carbonized at the same temperature and with/without activation—the nitrogen contents went from decreasing (carbonized at 900 °C) to increasing (carbonized at 1100 °C), which could be ascribed to the fact that the temperature of CO_2_ activation was lower than those of the carbonization temperature. The results indicated that the carbonization temperature played an important role in controlling the nitrogen content of the ACNFs.

### 3.3. Fourier Transform Infrared (FTIR) Spectroscopy

**Figure 2:**
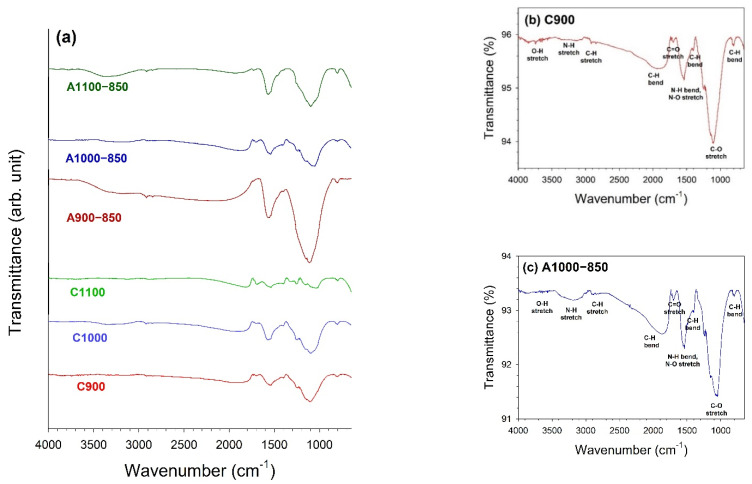
The FTIR spectra of the samples. (**a**) All samples, (**b**) C900, (**c**) A1000−850.

The FTIR spectra of the samples are shown in Figure 2a, and the patterns of C900 and A1000−850 are highlighted in Figure 2b,c, respectively. The bands in the regions 3000–2800 cm^−1^, 2000–1800 cm^−1^, 1410–1395 cm^−1^, and 820–790 cm^−1^ were assigned to the vibrations of aliphatic C–H groups. The strong band at 1105 cm^−1^ was attributed to C–O stretching vibrations. The characteristic absorbance peak at 1534 cm^−1^ was due to the N–H bending and N–O stretching vibrations. The bands at 3400–3200 cm^−1^ and 4000–3600 cm^−1^ were assigned to N–H and O–H stretching vibrations, respectively [56,57,58]. As seen in Figure 2a, the strength of the absorbance band at 1534 cm^−1^ was highly associated with the nitrogen contents (Table 1). Moreover, the increase in the number of defect sites of the graphite-like structure due to the incorporation of nitrogen atoms into the carbon lattices may result in the existing bands in the FTIR continuously shifting to higher frequencies (wavenumbers) [59].

### 3.4. X-ray Photoelectron Spectroscopy (XPS)

The XPS was carried out to investigate the surface elemental compositions and their chemical states on the surfaces of the samples. The XPS survey scan spectra of the samples revealed that there were three major peaks, which were caused by the C 1 s, N 1 s, and O 1 s photoelectrons. The atomic ratios of C 1 s, N 1 s, and O 1 s on the surfaces are summarized in Table 1. Although the results of EA indicate the contents in the bulk phase and XPS data provide surface compositions, the trends for the nitrogen contents were very similar. The deconvolution of high-resolution XPS spectra over the C1s, O1s, and N1s regions for all samples was performed in order to comprehend the chemical bonding states. 

**Figure 3:**
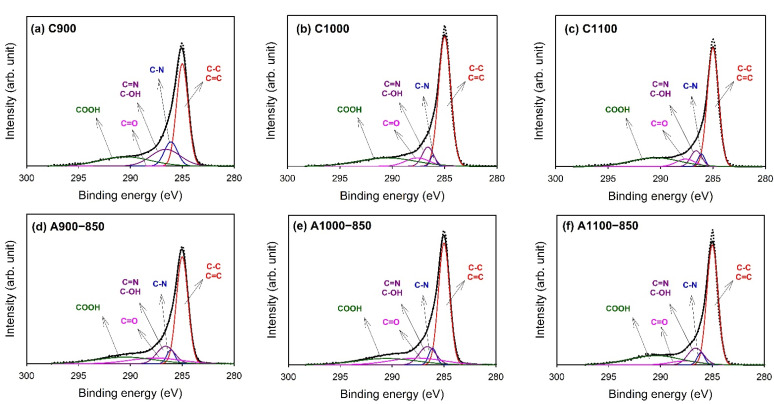
X-ray photoelectron spectroscopy (XPS) C 1s spectra: (**a**) C900; (**b**) C1000; (**c**) C1100; (**d**) A900−850; (**e**) A1000−850; and (**f**) A1100−850. The values are given in the atomic ratio (%) of total intensity.

Figure 3 shows the high-resolution XPS spectra of the samples in the C 1s binding energy region. The profiles of the XPS C 1s peaks were not significantly changed whether the samples were carbonized at different temperatures or further activated. Table 2 shows the calculated percentages of non-functional and functional carbon atoms. The C 1s spectra were decomposed into at most five identified components that represented carbon atoms due to aliphatic and aromatic backbones (C–C/C=C) at 285 eV and carbon atoms present in C–N (286.1 eV), C=N/C–OH (286.6 eV), C=O (287.6 eV), and COOH (290.5 eV) [60,61,62,63]. The C–C and C=C were the predominant groups for all samples. C900 contained much higher percentages of nitrogen-containing functional groups. However, when the carbonization temperature increased, the nitrogen groups significantly decreased. It is worth noting that CO_2_ activation contributed to the increase in the percentages of C=N, C–OH, and C=O. In addition, the percent of COOH in the samples remained very stable regardless of the treatment applied.

**Figure 4:**
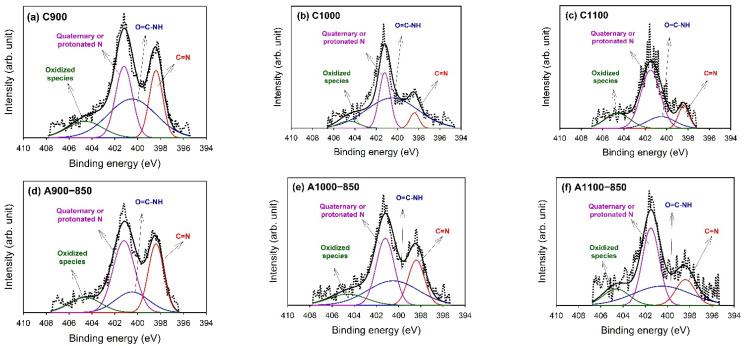
X-ray photoelectron spectroscopy (XPS) N 1 s spectra: (**a**) C900; (**b**) C1000; (**c**) C1100; (**d**) A900−850; (**e**) A1000−850; and (**f**) A1100−850. The values are given in an atomic ratio (%) of total intensity.

The results of the fits of the XPS N 1s spectra [63,64,65,66] are illustrated in Figure 4. The spectra demonstrate that carbonization and activation had evident influences on the N 1 s peaks, which are characterized by a bimodal pattern. As seen in Figure 4, the peak at 398.4 eV was less stable than the other one at 401.2 eV. That means that functional groups with lower binding energies had a higher tendency to unbind from the surface when the samples were treated at high temperatures. The calculated percentages of functional nitrogen atoms are presented in Table 3. Four functional groups at 398.4, 400.5, 401.2, and 404.6 eV were resolved; these can be assigned to C=N, O=C–NH, quaternary or protonated N, and oxidized species. For the samples of C1100 and A1100–850, the quaternary or protonated N was shifted to 401.5 eV. No peak for NH_2_ (399–399.3 eV) was resolved. The major functionalities of most samples were O=C–NH and quaternary or protonated N, except for A900−850, which possessed a higher percentage of C=N groups. The quaternary or protonated N was believed to be caused by the precursor of the carbon nanofibers (PAN).

**Figure 5:**
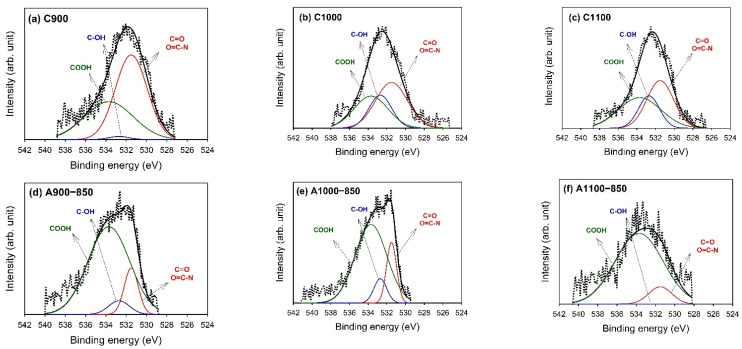
X-ray photoelectron spectroscopy (XPS) O 1 s spectra: (**a**) C900; (**b**) C1000; (**c**) C1100; (**d**) A900−850; (**e**) A1000−850; and (**f**) A1100–850. The values are given in an atomic ratio (%) of total intensity.

Additional information on the nature of surface oxygen-containing functional groups might be provided from the deconvolution of the XPS O 1 s spectra (Figure 5). Compared with the XPS N 1 s peaks, the effect of treatments on the XPS O 1 s spectra represented different patterns. The carbonization temperature only had a subtle effect on the shape of the spectra; however, the CO_2_ activation generated many more oxygen-containing functional groups at higher binding energies. Table 4 displays the atomic percentages of oxygen-containing functional groups [61,62,67]. The fitting revealed the presence of three surface oxides, including the C=O/O=C–N (531.5 eV), C–OH (532.7 eV), and COOH (533.7 eV) groups. As seen from the data, the major oxygen groups on the carbonized samples were the C=O/O=C–N and COOH groups. However, the C=O/O=C–N and C–OH groups were released significantly after activation; hence, the primary oxygen groups on activated samples were only COOH groups.

### 3.5. N_2_ Adsorption–Desorption Isotherms

**Figure 6:**
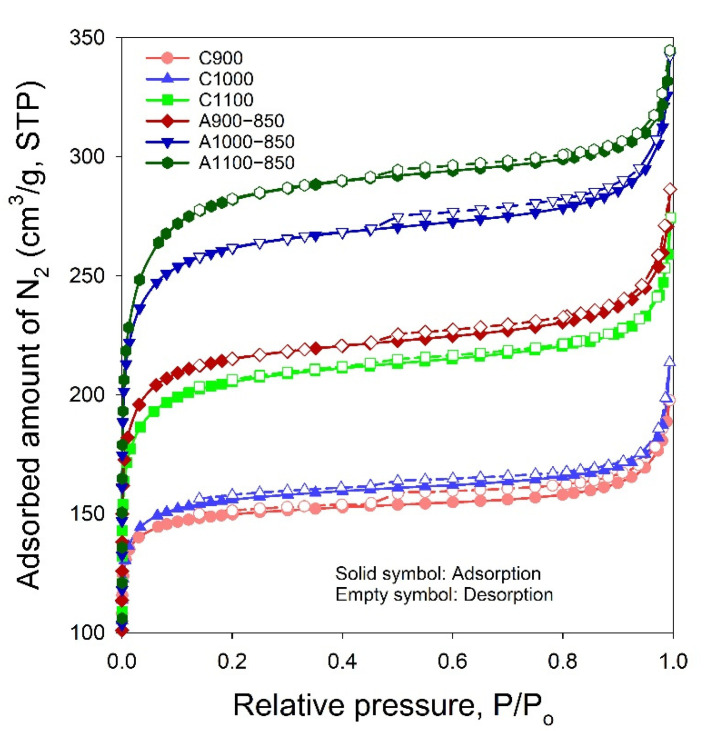
N_2_ adsorption–desorption isotherms at −196 °C for all samples.

The N_2_ adsorption–desorption isotherms of the samples at −196 °C are shown in Figure 6, in which the carbonized and activated samples are given for comparison. Regardless of whether the samples were carbonized or activated, the N_2_ adsorption isotherms were essentially type I according to the Brunauer–Emmett–Teller (BET) classification. There were no significant hysteresis loops, which indicated their microporous character. Capillary condensation was observed at relative pressures approaching 1.0, which could be due to the space where the nanofibers were intertwined. As seen from Figure 4, the N_2_ adsorption amounts were proportional to the carbonization temperature. Moreover, the N_2_ adsorption amounts were considerably improved after activation. 

The surface structure properties of the samples determined from the N_2_ adsorption–desorption isotherms are shown in Table 5. The BET specific surface area (SSA), micropore surface area (S_mi_), total pore volume (V_t_), and micropore volume (V_mi_) were increased with respect to the carbonization temperatures. Moreover, the subsequent activation could conspicuously improve these properties. However, the ultramicropore (pore size less than 0.7 nm) volumes showed a different trend. Except for the ratio of V_mi_ to V_t_, the ratios of S_mi_/SSA, V_<0.7 nm_/V_t_, and V_< 0.7 nm_/V_mi_ were decreased with increasing carbonization temperatures. This suggested that a higher carbonization temperature would lead to a lower microporosity for the HACNFs. There was a significant reduction in the ratios of V_<0.7 nm_/V_t_ and V_<0.7 nm_/V_mi_ after activation. It was believed that CO_2_ activation could partially oxidize the carbon atoms and that these carbon oxides might evaporate, thus generating more micropores and inducing pore enlargement. This could be proven from the median pore width shown in Table 5. Therefore, the pore volumes increased but the microporosity fell off.

**Figure 7:**
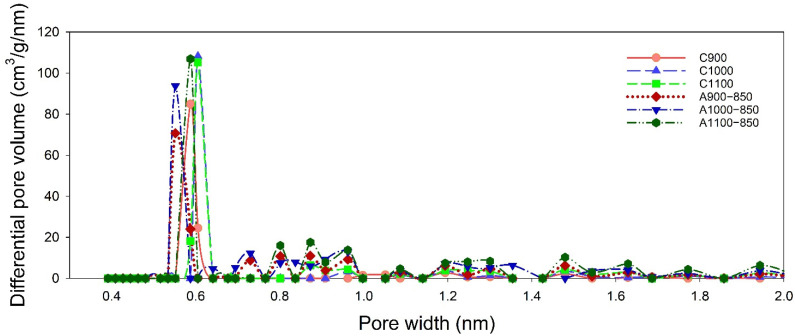
Pore size distributions of the samples, derived from the N_2_ adsorption isotherms at −196 °C by the NLDFT model.

Figure 7 illustrates the pore size distributions in the 0.4–2 nm range identified from the HS−2D−NLDFT model. The main micropores for all samples were at approximately 0.55–0.65 nm. The activation of C900 and 1000 enabled the pore size distribution to extend to smaller pore sizes. The pore size distributions shown in Figure 7 echoed the results shown in Table 5.

### 3.6. CO_2_ Adsorption

**Figure 8:**
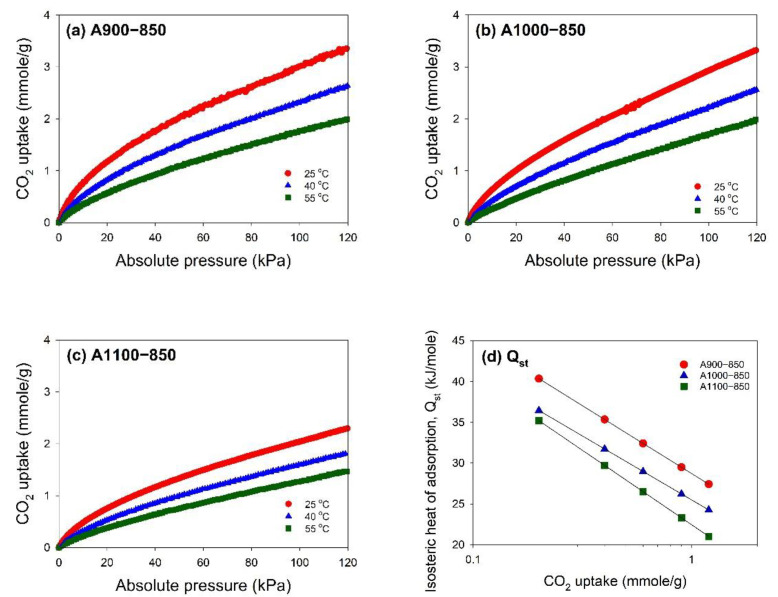
Adsorption isotherms of CO_2_ of the samples: (**a**) A900−850; (**b**) A1000−850; (**c**) A1100−850; and (**d**) the isosteric heat of adsorption of CO_2_ on the samples.

Figure 8 displays the adsorption isotherms of CO_2_ on the HACNFs under a pressure less than 123 kPa at different temperatures (25, 40, or 55 °C). For each measurement point, an equilibrium time of 45 s was maintained after achieving the set pressure value. As seen from Figure 8a–c, the CO_2_ uptakes increased with increasing pressure and decreasing temperature. The temperature dependence of the CO_2_ adsorption capacity in the range from 25 to 55 °C on the HACNF samples was consistent with the features of the exothermic reactions. The adsorption performance of CO_2_ followed the order A900−850 (3.03 mmol/g) > A1000−850 (2.96 mmol/g) > A1100−850 (2.06 mmol/g) at 25 °C and 1 atm. At 25 °C and 0.15 atm (a typical untreated flue gas composition), the CO_2_ adsorption capacities had the same order—i.e., A900−850 (0.99 mmol/g) > A1000−850 (0.84 mmol/g) > A1100−850 (0.63 mmol/g). 

It is generally believed that the Lewis-base on the surface of adsorbents could improve their CO_2_ uptakes, as CO_2_ is regarded as a weak Lewis acidic gas. In addition, the electron-donor properties induced by the nitrogen-containing functional groups could attract the CO_2_ molecules towards the adsorbents [7,68,69,70]. Specifically, the imines and amines groups are usually regarded as the Lewis base and could generate distinct interactions with CO_2_ molecules. Among the activated samples in this study, A900−850 featured the highest bulk nitrogen content (6.8 wt.%) and surface nitrogen groups (5.4 at.%). As seen in Table 3, the atomic percentages of N=C (imines or pyridine-type N) and quaternary or protonated N (amines) groups on the activated samples were highly associated with their CO_2_ uptakes. These two functional groups could be responsible for the high CO_2_ adsorption capacities of A900−850. The nitrogen atom in pyridonic N (O = C–NH) is believed to be surrounded with a higher electron density and behave as a strong Lewis base [69]. However, it was found to be reactive during the CO_2_ activation process. Therefore, it could not be the essential functional groups.

Figure 8d shows the variation in the Q_st_ values with the CO_2_ loading, in which the Q_st_ values log-linearly decreased with the CO_2_ loading from 0.2 to 1.2 mmol/g, indicating that the adsorption active sites on the surface of HACNFs were energetically heterogeneous for CO_2_ capture [71]. The CO_2_ adsorption on HACNFs was assigned to a typical physical adsorption according to Q_st_ values lower than 40 kJ/mol. The interactions between CO_2_ molecules and the nitrogen-containing functional groups or the enhanced micropore confinement [72] could be responsible for the higher Q_st_ values at a lower CO_2_ loading. Absolute values of the slope in the log-linear plot followed the order A1100−850 > A900−850 > A1000−850. This implied that the values of adsorption enthalpy were closer at low CO_2_ loadings for all samples, where the surface chemistry or ultramicroporosity controlled the adsorption performance, while the enthalpy decreased gradually when the CO_2_ loading grew, where the pore sizes might be the predominant factor affecting the CO_2_ uptakes.

In this study, the Freundlich equation was adopted to curve-fit the equilibrium adsorption data; the results are plotted in Figure 8a–c and summarized in Table 6. As seen from the data, the values of $K_{F}$ varied inversely proportional to the adsorption temperature as well as the carbonization temperature. However, the values of parameter *n* presented a different character. The values of *n* still decreased with increasing adsorption temperatures, but the least values of *n* occurred at A1000−850, which was responsible for the lowest slope obtained in the discussion about $Q_{st}$. Nevertheless, the values of *n* ranged from 1.69 to 1.24 and were indicative of feasible adsorption. Table 7 summarized the CO_2_ uptakes for the adsorbents in this study and various support materials using electrospinning in the literature, which implied that the CO_2_ adsorption for the HACNFs prepared in this study were comparable or superior to those of other ACNFs.

**Figure 9:**
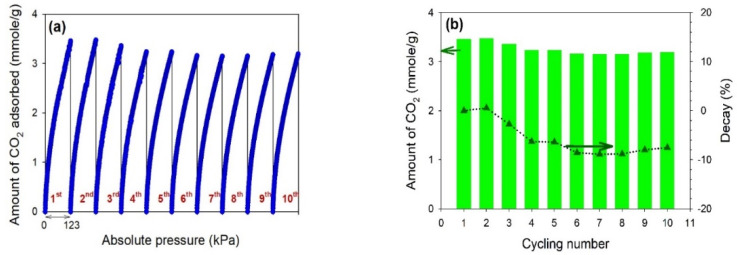
(**a**) Successive 10 cyclic adsorption tests and (**b**) the variation in the CO_2_ adsorption capacities at 123 kPa.

The cyclic regeneration of the adsorbents is an important property for evaluating their potential industrial applications. Therefore, 10 cyclic runs of the CO_2_ adsorption–desorption process were carried out on the adsorbent A900−850. Figure 9a shows the ten-cycle results of the adsorption–desorption experiments for the CO_2_ at 25 °C. Within each cycle, the adsorbents were desorbed by subjecting to the conditions of a dynamic vacuum. The variation in the adsorption capacities for CO_2_ at A900−850 are displayed in Figure 9b, and the degradation of the CO_2_ uptakes after 10 cyclic runs could be held within 8.9%.

## 4. Conclusions

Coaxial electrospinning followed by stabilization, carbonization, and activation has been successfully used to fabricate HACNFs with a hollow tubular structure. The HACNFs were smooth on the surface and inside the wall, with outer diameters ranging from 550 to 750 nm and a shell thickness of 75 nm. The carbonization temperature played an important role in controlling the nitrogen content in the HACNFs. The interpretation of the FTIR spectra sometimes encountered some difficulties in the identification of nitrogen functional groups, but the XPS could take over to perform this work. The activated samples carbonized at 900 °C featured the highest bulk nitrogen content (6.8 wt.%) and surface nitrogen groups (5.4 at.%). HACNFs are microporous adsorbents. The increase in the carbonization temperature (>900 °C) or CO_2_ activation was capable of increasing the specific surface areas or pore volumes; however, the effect of the pore enlargement induced from the above stages would weaken the microporosity. CO_2_ adsorption on HACNFs was an exothermic reaction, and belonged to the physical adsorption. The CO_2_ adsorption performance on the HACNFs and their cyclic regeneration tests demonstrated that the HACNFs should be promising candidates as gas adsorbents.

## Figures and Tables

**Table 1:** Elemental compositions from the elemental analysis (wt.%) and XPS analysis (at.%).

Sample	Elemental Analysis (wt.%)				XPS Analysis (at.%)		
	C	H	N	Others	C 1 s	N 1 s	O 1 s
C900	73.4	2.2	8.0	16.5	89.6	7.2	3.3
C1000	78.4	2.2	3.6	15.8	93.2	3.3	3.6
C1100	65.8	3.6	1.5	29.1	95.3	1.7	3.0
A900−850	68.6	2.8	6.8	21.8	91.4	5.4	3.2
A1000−850	64.6	3.5	3.6	28.2	92.6	3.1	4.3
A1100−850	70.4	2.9	2.8	23.9	96.6	2.0	1.4

**Table 2:** Results of the fits of the XPS C 1s region, values given in at.% (percent of total intensity).

Binding Energy (eV)	Type	C900	C1000	C1100	A900−850	A1000−850	A1100−850
285	C-C/C=C	43.2	58.0	58.8	48.2	49.4	56.0
		(48)	(62)	(62)	(53)	(53)	(58)
286.1	C–N	11.6	2.2	3.8	4.4	4.6	3.8
		(13)	(2)	(4)	(5)	(5)	(4)
286.6	C=N/C–OH	17.9	8.0	7.7	12.0	11.0	11.2
		(20)	(9)	(8)	(13)	(12)	(12)
287.6	C=O	−	7.4	6.2	11.7	13.4	5.0
		(0)	(8)	(6)	(13)	(14)	(5)
290.5	-COOH	16.9	17.6	18.9	15.2	14.2	20.6
		(19)	(19)	(20)	(17)	(15)	(21)

**Table 3:** Results of the fits of the XPS N 1s region, values given in at.% (percent of total intensity).

Binding Energy (eV)	Type	C900	C1000	C1100	A900−850	A1000−850	A1100−850
398.4	N=C	1.4	0.2	0.2	1.5	0.6	0.3
		(20)	(6)	(12)	(28)	(20)	(15)
400.5	O=C–NH	2.9	2.1	0.3	1.0	1.1	0.7
		(41)	(63)	(16)	(18)	(35)	(33)
401.2	Quaternary or protonated N	1.91	0.83	0.93	2.24	1.04	0.79
		(27)	(25)	(55)	(41)	(33)	(39)
404.6	Oxidized species	0.9	0.2	0.3	0.7	0.4	0.3
		(13)	(6)	(18)	(13)	(12)	(13)

**Table 4:** Results of the fits of the XPS O 1 s region, values given in at% (percent of total intensity).

Binding Energy (eV)	Type	C900	C1000	C1100	A900−850	A1000−850	A1100−850
531.5	C=O/O=C–N	1.3	1.6	1.2	0.5	0.8	0.1
		(38)	(44)	(39)	(14)	(18)	(11)
532.7	C–OH	0.3	0.8	0.6	0.2	0.4	−
		(9)	(23)	(21)	(6)	(9)	(0)
533.7	COOH	1.7	1.2	1.2	2.5	3.1	1.3
		(53)	(33)	(40)	(80)	(73)	(89)

**Table 5:** Surface features of the samples determined from the N_2_ adsorption–desorption isotherms at −196 °C.

Sample	SSA ^α^ (m^2^/g)	S_mi_^β^ (m^2^/g)	V_t_ ^γ^ (cm^3^/g)	V_ma_ ^φ^ (cm^3^/g)	V_me_ ^η^ (cm^3^/g)	V_mi_ ^η^ (cm^3^/g)	V_< 0.7 nm_ ^η^ (cm^3^/g)	d_HK_^ξ^ (nm)	S_mi_/SSA	V_mi_/V_t_	V_<0.7 nm_/V_t_	V_<0.7 nm_/V_mi_
C900	502	434	0.3057	0.0192	0.0497	0.2368	0.2170	1.35	0.86	0.77	0.71	0.92
C1000	524	441	0.3303	0.0379	0.0518	0.2406	0.2059	1.43	0.84	0.73	0.62	0.86
C1100	696	558	0.4244	0.0458	0.0631	0.3155	0.2360	1.50	0.80	0.74	0.56	0.75
A900−850	725	597	0.4429	0.0360	0.0695	0.3374	0.1979	1.49	0.82	0.76	0.45	0.59
A1000−850	883	714	0.5297	0.0428	0.0783	0.4086	0.2185	1.55	0.81	0.77	0.41	0.53
A1100−850	954	734	0.5331	0.0428	0.0563	0.4340	0.2148	1.62	0.77	0.81	0.40	0.49

^α^ SSA (specific surface area) was determined by the BET method. **^β^** S_mi_ was determined by the t-plot method. **^γ^** V_t_ represented the single point total pore volume at P/P_o_ ≈ 0.99. ^η^ V_me_, V_mi_, and V_< 0.7 nm_ were determined by a non-local density functional theory (NLDFT) model. ^φ^ V_ma_ was obtained by subtraction. ^ξ^ d_HK_ was the median pore width obtained by the Horváth–Kawazoe (HK) method.

**Table 6:** Fitted results of Freundlich equation for the CO_2_ adsorption data for the samples at different temperatures.

Sample	Temperature (°C)	K_F_ (mmol/g/kPa^1/n^)	n	R^2^
A900−850	25	0.1988	1.69	0.9994
	40	0.1186	1.55	0.9997
	55	0.0700	1.43	0.9999
A1000−850	25	0.1333	1.49	0.9997
	40	0.0791	1.38	0.9998
	55	0.0414	1.24	0.9999
A1100−850	25	0.1166	1.61	0.9998
	40	0.0666	1.45	0.9998
	55	0.0385	1.31	0.9999

**Table 7:** Comparisons of CO_2_ uptakes on the adsorbents in this study with various support materials using electrospinning in the literature.

Adsorbent	Precursor	S_BET_ (m^2^/g)	Conc. of CO_2_	Temp. (°C)	CO_2_ Uptake (mmol/g)	Reference
Hollow ACNF	PAN	725	1 atm 0.15 atm	25	3.03 0.99	This study
Hollow ACNF	PAN	884	1 atm 0.15 atm	25	3.16 0.94	[73]
ACNF	PAN	362	1 bar	25	2.37	[50]
ACNF	PAN	872	1 atm 0.15 atm	25	3.47 1.01	[74]
ACNF	PAN	897	1 atm 0.15 atm	25	3.17 1.00	[75]
ACNF	PAN	486	1 bar 0.15 bar	25	2.25 1.09	[76]
ACNF	PAN, polyvinylidene fluoride	925	1 bar	25	2.21	[77]
ACNF	PAN	412	1 bar	25	0.92	[78]
ACNF	PAN Melamine	547	1 bar	25	1.44	[78]

## Data Availability

The data presented in this study are available on request from the corresponding author. The data are not publicly available due to privacy restrictions.
